# Overview of Ayurveda and Ashwagandha: Bioactive Phytochemicals and Potential Applications to Gliomas

**DOI:** 10.3390/molecules30214272

**Published:** 2025-11-03

**Authors:** Herbert B. Newton

**Affiliations:** 1 Neuro-Oncology Center and Brain Tumor Institute, University Hospitals of Cleveland Medical Center, Seidman Cancer Center, Cleveland, OH 44106, USA; herbert.newton@uhhospitals.org; Tel.: +1-216-844-3951; Fax: +1-216-983-0792; 2 Molecular Oncology Program, Case Comprehensive Cancer Center, School of Medicine, Case Western Reserve University, Cleveland, OH 44106, USA

**Keywords:** ayurveda, dosha, herbs, ashwagandha, withanolides, withaferin-A, withanone, glioblastoma, glioma, cancer

## Abstract

Ayurveda is the traditional medical system of India and has been in use for more than 5000 years. The focus of Ayurveda is to maintain harmony and balance of the three Doshas (*Vata*, *Pitta*, *Kapha*), or life forces, that govern the physiology and health of each individual. Ashwagandha is considered one of the most useful plants in the Ayurvedic system for various illnesses, including cancer. Ethnopharmacological and phytochemical analyses have been elucidating the bioactive compounds in ashwagandha that mediate the anti-cancer effects. The most bioactive compounds appear to be the withanolides, including withaferin-A (WFA), withanone, and other withanolide derivatives. The focus of this review will be to discuss the pre-clinical and translational anti-cancer properties of WFA, withanone, and selected withanolides in terms of their ability to inhibit the growth of systemic forms of cancer and gliomas. The mechanisms of action of how these compounds affect tumor cell growth will also be discussed in detail, and include the induction of apoptosis, the inhibition of signal transduction pathways, the arrest of the cell cycle, and the inhibition of receptor tyrosine kinases. The final part will review how ashwagandha and its bioactive compounds could be applied to glioblastoma and gliomas.

## 1. Introduction

### 1.1. Ayurveda: Overview of Concepts and Philosophy

Ayurveda is the ancient healing system of India and has been in practice for over 5000 years [1,2]. The written history of Ayurveda can be traced back to the “Vedic” phase of Indian history—from 1200 to 800 BC—when Sanskrit was the main language used in communication and teaching. In Sanskrit, Ayurveda is a combination of the words “*ayur*” (life) and “*veda*” (science or knowledge)—literally, the “science of life”. In Ayurvedic philosophy, there is a fundamental connection between the macrocosm and the microcosm, with human beings being a minute representation of the universe, containing within them everything that makes up the surrounding world [1,2]. The cosmos consists of the five basic elements: *Vayu* (air), *Teja* (fire), *Aap* (water), *Prithvi* (earth), and *Akasha* (space). The basic elements combine in pairs to create the three “Doshas” (*Vata*, *Pitta*, *Kapha*), or life forces, which govern the physiology of each individual [1,2]. The Doshas, along with the seven *dhatus* (tissues) and three *malas* (waste products), make up the human body. When the Doshas are in harmony or equilibrium, health is maintained. However, an imbalance (i.e., an increase or decrease in one or more of the Doshas) will lead to the manifestation of disease.

*Vata* Dosha is a combination of the elements of air and space, and means “wind” in Sanskrit [1,2]. It is the principle of kinetic energy and is responsible for all body movement and nervous system functions. It is located in the colon and large intestine (principal site), as well as in the entire nervous system and other locations. *Pitta* Dosha is a combination of fire and water, and means “bile” or “yellow” in Sanskrit [1,2]. It governs enzymes and hormones, and is responsible for digestion, pigmentation, body temperature, cellular metabolism, hunger, thirst, and mental activity. It is located in the stomach (principal site), as well as in the small intestines and other sites. *Kapha* Dosha is a combination of the elements of earth and water, and means “phlegm” in Sanskrit [1,2]. It connotes the principles of cohesion and stability, and regulates *Vata* and *Pitta*. *Kapha* is responsible for keeping the body lubricated and maintaining its solid nature, tissues, sexual potency, and strength. It is located in the lungs (principal site), thorax, and pleural cavity, as well as the head and neck region and other sites.

The three Doshas form the constitution of a human at birth, in a particular combination—called *Prakriti*—which determines the physical and mental characteristics of that individual over the entire lifetime [1,2]. There are seven basic types of *Prakriti*: *Vata*, *Pitta*, *Kapha*, *Vata*/*Pitta*, *Pitta*/*Kapha*, *Vata*/*Kapha*, and *Vata*/*Pitta*/*Kapha* (triple balanced; rare). For most people, one Dosha will be predominant. *Vata* predominance leads to an “ectomorphic” constitution (i.e., light and thin build; anxious and fearful), *Pitta* types have a “mesomorphic” constitution (moderate build; aggressive and impatient), while *Kapha* individuals have an “endomorphic” constitution (solid, heavier build; tranquil and steady). When the Doshas lose their harmony and are out of balance (i.e., one or more Doshas become increased or decreased in proportion), an unhealthy condition or disease state can occur—called *Vikriti*. The Ayurvedic physician must then make the proper assessment, diagnosis, and Ayurvedic treatment plan to restore balance to the Doshas and induce resolution to the *Vikriti* condition.

For this review article, the available literature in English was accessed in PubMed using search terms including Ayurveda, ethnopharmacology, ashwagandha, withaferin-A, withanolides, glioblastoma, glioma, cancer, and herbal treatment. The literature search was very broad and was not limited by any specific inclusion or exclusion criteria; there were no biases present in the search. Since there have not been any published human clinical trials applying Ayurveda, ashwagandha, or associated phytochemicals in brain tumor patients, the focus of this review will be on pre-clinical in vitro and in vivo tissue culture and animal studies.

### 1.2. Medicinal Plants and Ethnopharmacology

Medicinal plants and herbs have been used for the therapy of human disease for thousands of years in many forms of traditional medicine [1,2,3,4]. Ayurvedic texts have descriptions of hundreds of herbs and thousands of herbal formulations that can be applied to many different illnesses. Some of the plants and herbs that are frequently prescribed in Ayurveda include ashwagandha, curcumin, fenugreek, pipalli, guduchi, amalaka, bramhi, and many others. In the treatment of many patients, the herbs are not used as individual compounds, but instead are administered as part of a recipe, mixture, or formulation called a “Rasayanas” [2,4]. These recipes of multiple herbs contain many potentially bioactive and interactive compounds with the potential to target various biochemical and cellular pathways in the tissues all at once. One of the most frequently prescribed plants in the Ayurvedic system is ashwagandha, which is classified as the genus *Withania* and species *somnifera*. There are also two recognized subspecies—*Withania somnifera Dunal* and *Withania somnifera Kaul*. Ashwagandha has recently been subjected to “Western medicine” style biochemical and molecular analyses to discern the bioactive components.

The new discipline of ethnopharmacology evolved in the 1800s, and was predicated on studying and analyzing the plants, recipes, and treatment approaches of ethnic medicine [5]. The ethnopharmacological investigation of medicinal plants and phytochemicals has been critical for drug discovery programs in many areas, including infectious diseases, brain disorders (e.g., epilepsy, depression, dementia), anti-inflammatory treatments, metabolic conditions, and, most importantly, anti-cancer drugs [6,7]. As of 2017, of the more than 120 anti-cancer prescription drugs on the market, 90 were plant-derived, and many of them were discovered from “folklore” claims of efficacy. Once a plant has been recognized as “medicinally active”, it is evaluated using a “Western medicine” style phytochemical analysis to search for the bioactive compound or components. The plant is processed—including leaves, flowers, fruit, seeds, branches, stem, and roots—and then an initial extraction is undertaken of all the constituents in the plant tissue, using various procedures: e.g., methanol extraction, ethanol extraction, and organic extraction. The aqueous phase contains water-soluble molecules, including nucleic acids, while the organic phase contains the proteins and lipids. Once the molecules of interest have been separated and isolated, they are analyzed further depending on their classification: Northern blotting, Western blotting, proteomics analysis, Nuclear Magnetic Spectroscopy, RNA and DNA sequencing, and in vitro testing against various cell cultures, including a panel of cancer cells (lung, breast, colorectal, brain, etc.). It is also imperative for the phytochemical to be tested at the molecular level, to establish how it influences the cancer cell in terms of specific pathways, including various receptors (e.g., epidermal growth factor receptor), internal signal transduction pathways (e.g., Raf-Mek-Erk, PI3K, mTOR), apoptotic proteins, angiogenesis, DNA methylation, extracellular matrix invasion, and transcription. If the compounds have activity against glioma cells in a culture, then they should undergo further testing in animal models, most often mouse or rat models with implanted tumors into the subcutaneous tissues or the brain.

Overall, these analyses have been focused on isolating phytochemicals from Ayurvedic herbs, such as ashwagandha, and analyzing their mechanisms of action for drug discovery potential. Most of the studies have been reductionist and have not taken into account the fact that the majority of Ayurvedic treatment approaches prescribe multiple herbs in various combinations and concentrations. This has led to a new, broader pharma approach called “*Network Pharmacology*”—first described by Hopkins in 2007–2008—that does not adhere to the belief of “one drug for one target for one disease” [8,9]. Instead, it suggests that many herbs could act on multiple targets simultaneously, rather than on a single intended target. Network pharmacology incorporates systems biology, proteomics and other “-omics” technologies, and computational biology to study multi-component and multi-targeted formulations.

## 2. Analysis of Ashwagandha and Bioactive Phytochemicals

**Figure 1:**
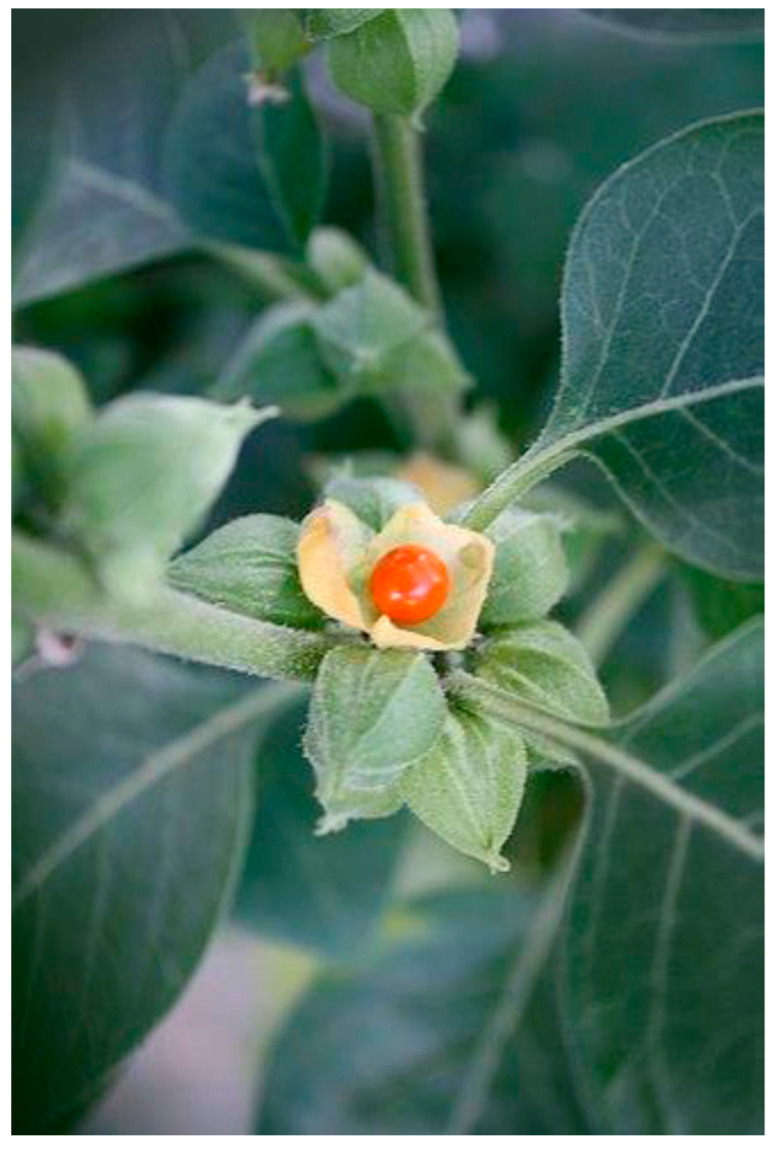
Ashwagandha plant, with flower and fruit [Adapted from Wikipedia, and used with the permission of Wowbobwow12 at English Wikipedia; Open Access].

Since its initial discovery and description in 6000 BC, Ashwagandha has been one of the most commonly used herbal medicines [8,10,11,12]. It is an evergreen woody shrub of the Solanaceae family that grows diffusely in the drier parts of India (see Figure 1); however, it is also grown specifically as a medicinal crop. The species name has been attributed as “*somnifera*”, which is equivalent to “sleep-inducer” in Latin, due to its ability to improve anxiety and stress. Within Ayurvedic practice, all of the different parts of the ashwagandha plant have medicinal applications and are used in various formulations, including the whole plant, leaves, roots, flowers, fruits, seeds, carbuncles, stems, and tubers; however, the roots are the most extensively prescribed. Over many years, ashwagandha has been reported to have numerous indications, including activity against inflammation, arthritis, stress, anxiety, depression, cardiac disease, epilepsy and other neurological disorders, GI health, hepatic disease, and pain syndromes, as well as for cancer. A partial list of some of the common ethnomedical uses of ashwagandha can be seen in Table 1.

It is only in the last 10 years that ashwagandha has been studied in the setting of “Western style” clinical trials [8,10,11,12,13]. Several randomized, double-blinded, placebo-controlled clinical trials have now been performed for various non-malignant conditions, including schizophrenia, STAR (strength training adaptations and recovery), idiopathic male infertility, subclinical hypothyroidism, mild cognitive impairment, and body weight management under chronic stress [14,15,16,17,18,19]. All of the studies demonstrated the significant effectiveness of ashwagandha for the treatment conditions in comparison to the control groups. In addition, the trials were all in agreement that the use of ashwagandha was safe and well tolerated in all of these different types of patients, at doses ranging from 200 mg/kg to 1000 mg/kg.

In contrast to the non-malignant diseases noted above, initial scientific interest in the application of ashwagandha to cancer started much earlier [8,10,11,12,13]. Chemicals were first isolated from ashwagandha leaves in the early 1960s and were found to have chemotherapeutic properties, followed by studies of root extracts in the 1970s that classified these chemicals as steroidal lactones [20,21]. However, it was not until the 1990s that ashwagandha was evaluated more formally in terms of its anti-cancer effects, including the ability to impede the growth of cancer cells, induce apoptosis by generating reactive oxygen species (ROS), and sensitize cancer cells to undergo apoptosis [10,11,12,13]. In addition, these early studies demonstrated that chemicals from ashwagandha (e.g., withaferin-A [WFA]) could work synergistically with traditional chemotherapy to inhibit tumor cells in tissue culture. More recent publications have focused on the anti-cancer potential of ashwagandha, reviewing the diverse groups of phytochemicals contained in the different parts of the plant, and how these various chemicals can affect growth, proliferation, motility, inflammation, the inhibition of apoptosis, and other neoplastic processes [8,11,13,22,23]. For example, aqueous root extracts of the plant have demonstrated the ability to modulate peripheral blood mononuclear cells and leukemic THP-1 cell viability, as well as increase oxidant scavenging and caspase (-8, -9, -3/-7) activities, while also decreasing tumor necrosis factor-alpha (TNF-α), interleukin-10 (IL-10), and glutathione levels. Crude water extracts from ashwagandha were also observed to modify pro-apoptotic and tumor-promoting proteins, thereby inhibiting tumor growth, including nuclear factor-kappa B (NF-κB), phospho-Akt, Bcl-xl, heat shock protein 70 (HSP70), cyclin D1, vascular endothelial growth factor (VEGF), matrix metalloproteinases, and others. Anti-neoplastic activity has been noted when ashwagandha and its preparations have been applied to numerous tumor types in vitro and in animal models, including lung cancer, breast cancer, prostate cancer, colorectal cancer, pancreatic cancer, renal cell carcinoma, ovarian cancer, gliomas, and others (see sections below).

### 2.1. Steroidal Lactones and Withanolides

**Figure 2:**
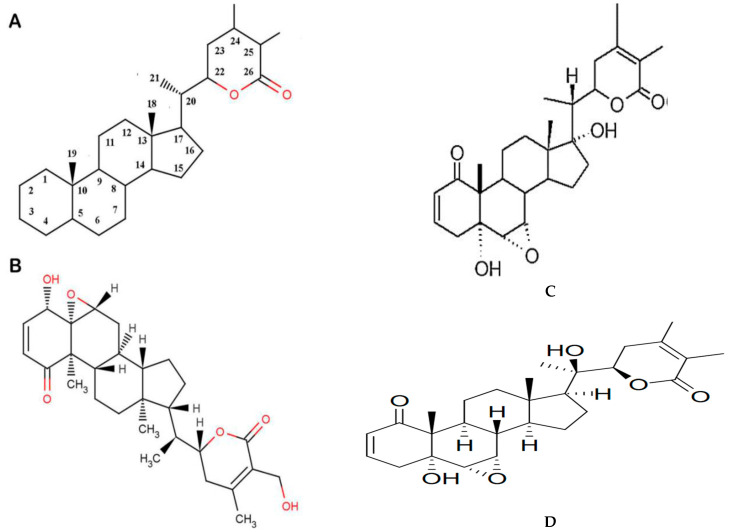
(**A**) Structure of the ergostane skeleton of withanolides. (**B**) Structure of withaferin-A. Adapted from Grover et al., with permission from the corresponding author. (**C**) Structure of withanone. Adapted from reference [27] and used with the permission of the corresponding author. (**D**) Structure of withanolide-A. Fully adapted from Wikimedia Commons; Open Access.

Numerous unique classes of bioactive phytochemicals have been isolated from ashwagandha, including steroidal lactones (e.g., withanolides, WFA), alkaloids (e.g., withanine), flavonoids (e.g., quercetin), steroids, salts, and nitrogen-containing compounds (e.g., withanol) [10,11,12,13,22]. A more complete list of the different classes of phytochemicals present in ashwagandha can be seen in Table 2. The phytochemical group with the most potent anti-cancer activity is the withanolides [24,25,26]. Withanolides are a naturally occurring class of C-28 steroidal lactones based on the ergostane skeleton (see Figure 2A) that are highly oxygenated and reactive at positions C-26 and C-22 or C-26 and C-23, forming δ- or γ-lactones. The withanolide family encompasses roughly 1200 members at this time, which are formed via ring fission, cyclization, or skeletal rearrangement of the steroid nucleus or the nine-carbon side chain. The most well-known and fully characterized withanolides include WFA, withanone (WTHN), and withanolides-A (WTHA), -E, -F, -G, -H, -I, -J, -K, -L, and -M. Withanolides can be categorized into two major groups based on the arrangement of their side chain, with Type-A including those with a δ-lactone or δ-lactol at C-22 and C-26, and Type-B comprising those with a γ-lactone, typically involving C-23 and C-26. In addition to the large group of naturally occurring withanolides from ashwagandha and related plants, the chemical structure of withanolides can serve as a scaffold allowing for the synthesis of analogs that potentially have an enhanced anti-cancer potency, bioavailability, and reduced toxicity [24,25]. Monosubstituted, disubstituted, and trisubstituted withanolide analogs have been synthesized, as well as others with fused ring structures that are currently under study and may have a more potent anti-cancer activity.

The withanolides with the most potent anti-cancer bioactivity include WFA, withanone, withanolide-A, withanolide-E, withanolide-D, physalin A, physalin B, 4β-hydroxywithanolide-E, withalongolide-A, tubocapsanolide-A, coagulansin-A, and Ixocarpalactone-A [24,25,26]. All of these compounds have demonstrated activity against cancer cells in vitro, including some in the low micromolar range—0.03 to 0.1 μM (e.g., WFA, withanolide-D, withanolide-E). The in vitro anti-cancer activity of withanolides has been noted against numerous cell lines, including lung, breast, melanoma, glioma, hepatocellular, endometrial, neuroblastoma, renal, lymphoma, thyroid, head and neck, colorectal, prostate, leukemia, sarcoma, pancreatic, ovarian, and many others [24,25,26]. In vivo applications of withanolides have also demonstrated bioactivity in many tumor types, including breast cancer (TNBC MDA-MB-231 cell xenografts), colorectal cancer (HCT116 colon cancer cell xenografts), non-small cell lung cancer (NSCLC H292 xenografts), ovarian cancer (A2780 cell xenografts), pancreatic cancer (Panc-1 cell xenografts), hepatocellular cancer (H22 cell xenografts), and others [24,25,26]. Several anti-cancer mechanisms associated with withanolides have been demonstrated thus far, and include the enhancement of apoptosis, enhancement of autophagy, stimulation of ferroptosis and paraptosis, inhibition of the cell cycle and induction of cell cycle arrest, reduced expression and activation of NF-κB, induction of cellular senescence, inhibition of invasion and metastasis, targeting of stem cell-associated proteins, immunomodulatory effects, inhibition of angiogenesis, suppressing activity of matrix metalloproteinases, and disrupting the cytoskeleton and microtubular network [24,25,26,28]. Withanolides have also been shown to inhibit and modulate the activity of numerous receptors and signal transduction pathways of cancer cells, which will be elucidated in more detail in the following sections.

As a group, withanolides are known to have poor bioavailability and to be very hydrophobic, which may limit their clinical applications [24,25,26]. In addition, they are so diffusely bioactive that there may be issues with systemic toxicity when applied to cancer patients. This has led to research into novel formulations for the delivery of the pure compounds to patients, including metallic nanoparticles (e.g., gold nanoparticles), nanoliposomes, and nanosponges (a type of spherical nanoparticle, usually synthesized from a carbon-based polymer, that can encapsulate and release various substances). In spite of these limitations, clinical trials of withanolides have begun as of 2012, with several trials initially performed in patients with breast cancer, using ashwagandha as a method to improve cancer-related fatigue and overall quality of life, demonstrating positive results [29,30]. Another phase I trial was in patients with advanced osteosarcoma that used WFA, and that did not result in any dose-limiting toxicity [31]. However, the authors did note that the bioavailability of WFA appeared to be low and proposed further phase II studies using a dose of 216 mg/day. Additional ongoing studies include a phase I/II trial in recurrent ovarian cancer (WFA; NCT05610735), a phase I trial in general cancer patients (ashwagandha; NCT00817752), a phase II trial for cancer-related cognitive dysfunction (ashwagandha; NCT04092647), and a phase I/II trial for osteosarcoma (ashwagandha and curcumin; NCT00689195).

### 2.2. Withaferin-A: Overview and Application to Systemic Tumors

The ethnopharmacological and phytochemical analyses of ashwagandha have established that the most bioactive compounds are the steroidal lactones—in particular, the withanolides [24,25,26]. The most bioactive compound within the withanolide class of chemicals, especially in terms of its anti-neoplastic activity, is WFA (see Figure 2B) [32,33,34,35,36,37,38,39]. Structural examinations of WFA have revealed three sites that are the most reactive and would be vulnerable to nucleophilic attack: the unsaturated A-ring at C3, the epoxide structure at position 5, and the C24 in the E ring [32,33,34,35,36,37,38,39]. These sites would have the ability to bind covalently to the cysteine residues of proteins via alkylation reactions, resulting in diminished activity of the target proteins. Withaferin-A has been tested against numerous systemic cancer cell lines in vitro and has demonstrated positive activity, including in cell cultures of GBM, lymphoma, neuroblastoma, renal cell, leukemia, hepatocellular, prostate, osteosarcoma, breast, colorectal, ovarian, multiple myeloma, cervical, endometrial, melanoma, and head and neck cancer [32,33,34,35,36,37,38,39,40,41,42]. The bioactivity against these cell lines was quite robust, with IC_50_ values ranging from 0.03 to 24 μM. In addition, when WFA was used in combination regimens (e.g., TRAIL, cisplatin, Doxorubicin, Etoposide), it was able to sensitize tumor cells and enhance growth suppression and apoptosis [32,33,34,35,36,37,38,39,40,41,42]. In non-small cell lung cancer cell lines, WFA was noted to induce autophagy and apoptosis, as well as stimulate the activity of reactive oxygen species (ROS), at low IC_50_ values [40,41,42]. WFA could also inhibit general lung cancer cell growth in a culture, inhibit the growth of lung cancer “cancer stem cells” (CSCs), and reduce the ability of cells to form spheroids. Some of the inhibitory activity seemed to be mediated through the downregulation of mTOR and STAT3 signaling. WFA was also able to inhibit the “epithelial to mesenchymal transition” (EMT) in cultured lung cancer cells via reduced cell adhesion, motility, migration, and invasion. The inhibition of the EMT seemed to involve the suppression of TGF-β1 and TNF-α signaling. The proliferation and migration of lung cancer cells was also thought to be inhibited by WFA via the increased expression of pro-apoptotic molecules such as p53 and Bax, while reducing the expression of Bcl-2, possibly through the downregulation of two onco-micro-RNA’s—miR-10b and miR-27a. Similar pre-clinical findings have been noted in ER+ and triple-negative breast cancer cell lines, with reduced tumor cell proliferation, cell cycle activity, migration, cell adhesion, motility, and invasion, as well as the ability to inhibit breast CSCs and reduce the ability of cells to make the EMT [32,33,34,35,36,37,38,39,43,44].

**Figure 3:**
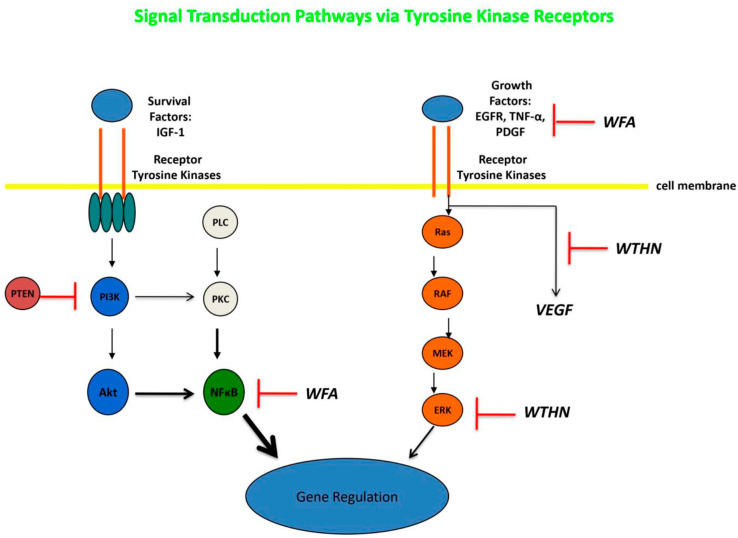
Diagram of signal transduction pathways, with WFA inhibiting NF-κB, EGFR, and TNF-α and WTHN inhibiting VEGF and ERK. Based on references [23,24,25,33,34,35,36,37]. WFA—withaferin-A; WTHN—withanone. Author’s own work.

The molecular mechanisms mediating the anti-neoplastic activity of WFA remain uncertain, but likely involve poly-pharmaceutical effects that target diffuse proteins and signal transduction pathways simultaneously. Withaferin-A has been shown to be a potent inhibitor of the ubiquitin-mediated proteasome pathway, which leads to the buildup of ubiquinated proteins within tumor cells, including Bax, p27, and IκBα. It has also been demonstrated to reduce cell proliferation by inducing cell cycle arrest at the G_2_/M phase, and can inhibit mitotic activity by upregulating phosphorylated Wee-1, phosphorylated histone H3, p21, and Aurora B targets (see Figure 3) [24,25,26,32,33,34,35,36,37,38,39]. Micromolar concentrations of WFA have also been noted to modulate and increase the redox potential inside of tumor cells, increasing oxidative stress and triggering cell death in many cell types. Withaferin-A is able to reduce the potential for metastasis by decreasing the expression of EMT markers, thereby inhibiting cellular motility, as well as by reducing the activity of the urokinase-type plasminogen activator (uPA) protease [24,25,26,32,33,34,35,36,37,38,39]. Cancer stem cells are typically very resistant to treatment and have the capacity for self-renewal. However, WFA has been demonstrated to modify CSCs so that they become more differentiated and sometimes can enter a senescent state. In vitro studies reveal that WFA can trigger apoptosis in tumor cells through several mechanisms, including reducing the activation of NF-κB by preventing the TNF-induced activation of IκB kinase β via a redox mechanism, as well as by upregulating tumor suppressor genes such as p53 and pRb [24,25,26,32,33,34,35,36,37,38,39]. Furthermore, WFA was able to induce death receptor-5 and transduce apoptosis signals, resulting in the apoptotic death of tumor cells. An additional pathway for WFA to mediate apoptosis is to increase Par-4 induction and p38 MAP kinase activation. In an immune competent tumor-bearing mouse model, WFA treatment was able to modify anti-tumor immunity via the inhibition of Treg proliferation and activation of apoptosis in Treg cells [32]. The application of WFA is also able to disrupt the cytoskeleton of cancer cells by covalently binding to annexin II, which impairs the ability of annexin II to bind with actin and induces the aggregation of actin. This disruption of the cytoskeleton is also related to the disassembly of vimentin, vimentin depolymerization, and filament aggregation [32,33,34,35]. Neoplastic cells are known to highly express heat shock proteins (e.g., HSP90), which act as molecular chaperones in proliferation, differentiation, invasion, and metastasis. WFA treatment was demonstrated to bind to HSP90 and disrupt the HSP90-Cdc37 complex, with the inhibition of complex activity and degradation of HSP90 target proteins [32].

The in vivo anti-cancer activity of WFA has been extensively evaluated in a variety of tumor types [24,25,26,32,33,34,35,36,37,38,39]. In non-small cell lung cancer (NSCLCA), A549 NSCLCA cell xenografts were established in mice and then treated with intraperitoneal injections of WFA 4 mg/kg or 8 mg/kg every other day, which resulted in a 60% and 53% growth inhibition, respectively [32,33,34,35,36,37,38,39,42]. In triple-negative breast cancer MDA-MB-231 mouse xenografts, WFA 4 mg/kg/day for 2.5, 5, and 28 days resulted in a tumor reduction by 80%, 45%, and 60%, respectively [31,32,33,34,42]. In addition, when using the 4T1 spontaneous mouse mammary carcinoma model, treatment with WFA at doses of 4 mg/kg and 2 mg/kg resulted in significantly reduced tumor growth rates compared to controls (*p* < 0.01), as well as a dose-dependent reduction in the number of metastatic lung nodules. In a colorectal mouse xenograft model using HCT116 colon cancer cells, treatment with WFA 2 mg/kg intraperitoneally every other day for 32 days resulted in significant reductions in tumor volume and weight, while the body weight of the animals remained unchanged [32,33,34,35,36,37,38,39]. Using ovarian cancer A2780 cells transplanted into mouse xenografts, WFA treatment (2 mg/kg) alone or in combination with cisplatin resulted in a reduction in tumor growth by 70% to 80% and a complete inhibition of metastasis to other organs in comparison to control animals. Withaferin-A treatment in prostate cancer mouse xenografts created using PC-3 or DU145 cells resulted in a 70% inhibition of tumor growth. Using nude mouse xenografts of pancreatic cancer Panc-1 tumor cells, treatment with WFA at 3 mg/kg or 6 mg/kg intraperitoneally resulted in a 30% and 58% reduction in tumor growth, respectively, without significant toxicity to the animals [32,33,34,35,36,37,38,39]. When WFA was used in combination with epirubicin and oxaliplatin against the pancreatic cancer xenografts, a synergistic increase in anti-tumor activity was noted.

The pharmacokinetic and pharmacodynamic profile of WFA has been evaluated in mouse models [32,34]. After an oral administration of WFA (1000 mg/kg), there is a very brisk oral absorption, and the peak plasma concentration is reached within 10 min. After an intraperitoneal injection of a single dose of WFA (4 mg/kg), a maximum plasma concentration up to 2 μM was reached, with a half-life of approximately 1.4 h.

### 2.3. Ashwagandha and Withaferin-A: Application to Gliomas

In the initial in vitro studies of WFA and brain tumor cells, the pure compound was not used. Instead, ashwagandha extracts from the leaves of the plant were applied to the cells [45,46,47]. The first study was in 2009, when Shah and co-workers used an alcoholic extract (i-Extract) of ashwagandha leaves in C6 (rat glioma) and YKG1 (human glioma) cell lines [45]. In addition to i-Extract, the cell lines also received individual components of the extract, including WFA, WTHA, and WTHN. All four of the treatment arms produced a substantial growth arrest for both cell lines in a dose-dependent manner, with a peak effect between 48 and 72 h. The IC_50_ values were lower for the human glioma cells in comparison to the rat glioma cells; WFA had the lowest IC_50_ for both cell lines: 0.1 μM and 0.2 μM, respectively. Higher doses of i-Extract, WFA, WTHA, and WTHN were noted to induce apoptosis in the glioma cell lines. However, low to moderate doses were able to induce cell cycle arrest in the S and G_2_/M phases, in addition to reducing motility and altering the phenotype to a more senescent state. In combination studies of the four compounds, WFA (0.01 μM) plus WTHN (5 μg/mL) was the most active in being able to induce a senescent and more differentiated state. The authors considered ashwagandha and its components as possible treatment options for differentiation therapy in glioblastoma. In a follow-up report from the same research team, they used the water extract from ashwagandha leaves and applied it to the rat C6 glioma cell line, as well as several human glioma cell lines—YKG1, U118MG, and A172 [46]. The preliminary phytochemical analysis of the aqueous extract noted the presence of flavonoids, steroids, tannins, amino acids, saponins, reducing sugars, and alkaloids. The extract was able to trigger apoptosis in all of the cell lines at higher concentrations (>1.0%), similar to the ethanol extract data noted above. At reduced concentrations (≤0.5%), there was an inhibition of cell proliferation, and all cell lines had impaired growth, with morphological changes that were more compatible with a senescent state (e.g., increased glial fibrillary protein [GFAP] expression, enlarged cell size, increased number of processes). In addition, the application of the aqueous extract also impaired cell motility and altered adhesion molecule expression, including neural adhesion molecule (NCAM) isoforms NCAM-120 and NCAM-140. Based on these data, the authors suggested that the aqueous extract of ashwagandha also had potential as a differentiation form of treatment for glioblastoma. In the final report from Kataria et al., they describe the use of the water extract of ashwagandha (ASH-WEX) against cell culture-derived C-6 glioma cells implanted into rat intracranial xenografts [47]. Treatment with daily oral ASH-WEX 0.5% resulted in a reduced tumor growth in comparison to control animals; the mean tumor volume was reduced by 43.61%, which was nearly significant in statistical testing (*p* < 0.057). In ASH-WEX-treated tumor cells, there was a dramatic reduction in the levels of pNFκB, pAkt, VEGF, cyclin D1, and BCL-XL, leading to a reduced growth and proliferation, as well as an enhanced apoptosis. Cell cycle analysis with flow cytometry detected a block and cell cycle arrest at G_0_/G_1_. Tumor cells were also found to have an upregulation of NCAM expression, with a reduced cell motility and invasiveness. Finally, in some animals with large tumors, after treatment with ASH-WEX and subsequent tumor shrinkage, there was some improvement in focal deficits (e.g., leg weakness, gait difficulty). See Table 3 for a summary listing of the applications of ashwagandha and its bioactive compounds to gliomas, including the relevant anti-cancer effects.

**Figure 4:**
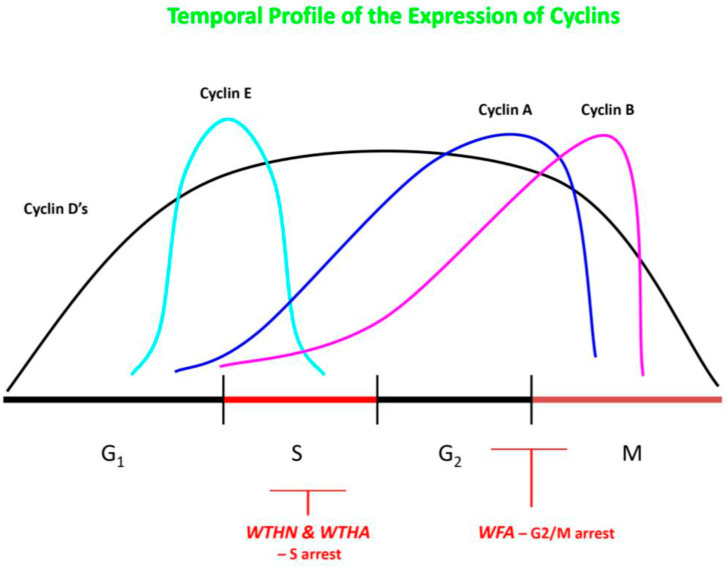
Diagram of WFA inhibiting the cell cycle at the G_2_/M phase, while WTHN and WTHA are inhibiting the cell cycle in the S phase. Based on references [23,24,25,33,34,35,36,37]. WFA—withaferin-A; WTHN—withanone; WTHA—withanolide-A. Author’s own work.

Since those initial studies, further applications of ashwagandha to brain tumors have focused on treatment with the major active constituent—WFA (see Figure 2B). After some early investigations using WFA isolated from the *Vassobia breviflora* plant, which is also in the Solanaceae family [48,49], Grogan and colleagues reported the results of their detailed investigation into the treatment of glioblastoma cell lines with WFA [50]. They used two human glioblastoma cell lines (U87, U251) and one murine cell line (GL26), and then applied varying concentrations of WFA. Each cell line demonstrated a dose-dependent shift in the cell cycle, with G_2_/M arrest, after exposure to WFA (see Figure 4). The WFA concentrations needed to attain peak G_2_/M arrest varied between cell lines and ranged from 0.5 μM to 1.5 μM. When the concentrations of WFA were elevated, an enhancement of apoptosis was documented in all cell lines, with reduced levels of uncleaved caspases, likely mediated via an activation of the extrinsic and intrinsic apoptotic pathways. Increasing concentrations of WFA also reduced the total levels of Akt and mTOR, along with lowered levels of phosphorylation in some cell lines. Application of WFA also resulted in reduced total levels of EGFR, Her2/ErbB2, and c-MET. In the GL26 and U87 cell cultures, the application of WFA resulted in an elevation of oxidation status, including peroxides and mitochondrial superoxide. A subsequent study by the same group applied WFA to glioblastoma cell lines that had become resistant to Temozolomide (TMZ), the “standard of care” chemotherapy drug for this tumor type [51]. TMZ-resistant sub-cultures of U251 and U87 were generated by exposing the parental lines to increasing concentrations of TMZ (30–300 μM) over 8 weeks. The subsequent WFA application to the resistant cell lines resulted in a reduction in cell proliferation and viability, with G_2_/M cell cycle arrest. At higher concentrations of WFA, there was evidence of increased apoptotic cell death through the intrinsic and extrinsic pathways. Similarly to TMZ non-resistant cells, the application of WFA resulted in decreased total levels of Akt and mTOR, as well as their phosphorylated counterparts. In addition, the overall levels of EGFR, Her2/ErbB2, and c-MET were reduced after treatment. Methylguanine methyltransferase (MGMT) is a very important enzyme in terms of mediating TMZ resistance in glioma cells. In the TMZ resistant cell lines, WFA treatment resulted in a significantly reduced concentration of MGMT, with a complete elimination at doses of 10 μM. The application of the combination of TMZ and WFA resulted in a synergistic effect on reducing MGMT, which allowed TMZ to regain efficacy against the cell lines. The authors concluded that future research in vitro and in animal models was important for investigating the use of WFA in combination with TMZ.

**Figure 5:**
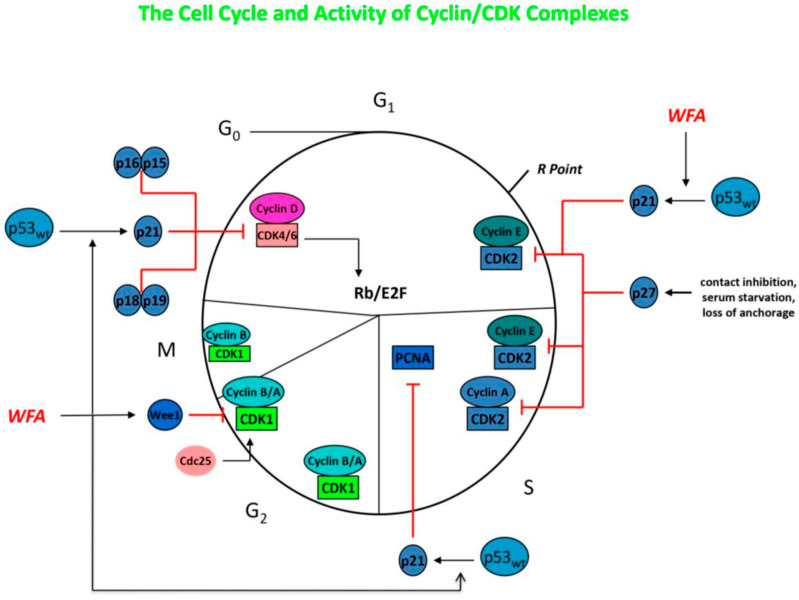
Diagram of the cell cycle and WFA activating p53 and p21, reducing activity of the cyclin D1/CDK4/6 complex, PCNA, and cyclin E/CDK2 complex. Based on references [23,24,25,33,34,35,36,37]. WFA—withaferin-A. Author’s own work.

Hou and colleagues replicated many of the findings noted in the studies reviewed above using rat C6 glioma cell lines [52]. Increasing concentrations of WFA were able to trigger a dose-dependent elevation in the number of cells undergoing apoptosis. This was correlated with an increased activity of caspase-3 and caspase-9. In addition, a dose-dependent increase in the expression of Bax was demonstrated, while the expression of Bcl2 was reduced. Furthermore, TNF-α expression was decreased after WFA treatment, accompanied by the inhibition of nuclear translocation of the NF-p65 subunit from the cytosol into the nucleus, thereby reducing NF-κB activation (see Figure 5).

Chang and co-workers have published several studies investigating the use of WFA, AshwaMAX (Pharmanza Herbal Pvt. Ltd. Gujarat, India), and tumor-treating fields (TTFs) on glioma cell lines and murine orthotopic brain tumor models [53,55,56]. AshwaMAX is a well-defined ashwagandha extract from the roots that is standardized to contain 4.3% or more of WFA, as well as 8.4% of total withanolides. In the first study, they used pure WFA and AshwaMAX for the treatment of patient-derived GBM2 and GBM39 cell lines and the commercial cell line U87 [55]. Increasing concentrations of WFA and AshwaMAX were able to inhibit growth and cell proliferation in all three cell lines. The IC_50_ values for WFA were in the submicromolar range—from 0.25 to 0.31 μM—while the IC_50_ values for AshwaMAX were higher and ranged from 2.1 to 14.8 μg/mL. The inhibitory activity was associated with a diminished ability of glioma cells to form neurospheres; any neurospheres that did form prior to WFA and AshwaMAX exposure were likely to collapse. Mouse xenografts using GBM2 and GBM39 cell lines were developed using Luciferase bioluminescent modified cells and then treated with AshwaMAX through oral gavage [52]. After treatment with AshwaMax, the bioluminescent signal was significantly reduced after one week and then had a stable reduced signal for another 3–4 weeks. However, after 4 or 5 weeks, the bioluminescent signal recovered towards the initial baseline, suggesting the outgrowth of an AshwaMAX-resistant cell clone. Overall, AshwaMAX mice survived an extra 4 weeks when compared to control treated mice.

In the second study, the Chang group also used GBM2, GBM39, and U87 glioma cell lines and treated the cells with WFA, TTFs, or a combination of both therapies [56]. Tumor-treating fields are an FDA-approved form of therapy for GBM that induce electrical fields in the tumor tissue, which are able to disrupt the movement and function of polarized proteins in the cells involved in mitosis (e.g., actin, septin), leading to metaphase, anaphase, and telophase arrest and apoptosis [57]. The exposure of the glioma cell lines to TTFs (4.0 V/cm) resulted in a significant and sustained growth suppression. When the U87 cells were treated with WFA at low doses (0.1 μM), growth was not significantly inhibited. However, when TTFs were applied after an exposure to low-dose WFA, growth inhibition was enhanced, with a substantial reduction in cell numbers. The exposure of glioma cells to WFA and TTFs in combination had a synergistic effect on the inhibition of tumor growth, along with a reduction in the size of the neurospheres and less adherent connectivity between the cells. In a review of WFA and its potential as a therapeutic approach for GBM and other cancers, the authors determined that significant supportive evidence was accumulating [53]. However, further pre-clinical, animal model, and human clinical trials were necessary for validation.

In a more recent publication, Tang and co-workers evaluated the response of U251 and U87 human glioma cell lines and nude mouse xenografts to treatment with WFA [58]. Similarly to the Chang study noted above, they documented a concentration and time-dependent inhibition of cell growth and an increase in apoptotic cell death, as well as significant increases in cleaved PARP1 and caspases -3, -7, and -9, without an increase in cleaved caspase-8. These results suggest that the WFA-mediated increase in apoptosis is being facilitated mainly through the intrinsic pathway. In addition, the intrinsic pathway was accelerated through the increased expression of Bim and Bad. They also confirmed cell cycle effects by documenting the arrest in G_2_/M induced by an upregulation of p21. The WFA-mediated disruption of apoptosis and cell cycle arrest were further evaluated using mRNA transcriptional analysis, which revealed that the ATF3-ATF4-CHOP axis may play a fundamental role. In U87 nude mouse xenografts, WFA treatment caused a substantial reduction in tumor growth and size in comparison to control animals.

A recent extensive review of the literature on pre-clinical data and molecular mechanisms of WFA, along with an analysis of its potential for the treatment of adult and pediatric brain tumors, was reported by Marlow and colleagues [59]. They discuss that WFA seems likely to adequately transfer through the blood–brain barrier (BBB) and passes Lipinski’s Rule of Five, since it has a relatively low molecular weight, has a favorable blood/brain partition coefficient, has only 2 hydrogen bond donors, and has less than 10 hydrogen bond acceptors. Withaferin-A also has activity against many of the pathways that are identified as being dysfunctional in high-grade gliomas, including the p53 signaling pathway, MAPK/ERK signaling pathway, PI3K/Akt/mTOR signaling pathway, NF-κB signaling pathway, and the angiogenesis pathways (e.g., elevated VEGF). The authors were in favor of recommending WFA for continued in vitro and animal studies, and to initiate early clinical trials in humans with GBM and other high-grade gliomas.

### 2.4. Withanone: Overview and Application to Systemic Tumors

Withanone is another steroidal lactone, with a C-28 chemical structure based on the ergostane skeleton, that is a member of the withanolide class of compounds (Type-A) [24,25,26,60]. The chemical structure of WTHN is similar to WFA but contains C6 and C7 epoxy groups, as well as hydroxyl groups at C5 and C17 (see Figure 2C). Although the literature for WTHN is much less extensive than for WFA, there is still considerable evidence that WTHN can inhibit numerous cellular pathways that are involved in neoplastic transformation and progression. For example, WTHN has been shown to disrupt the TPX2-Aurora A complex, which is important for spindle formation and the reorganization of interphase microtubules during the mitosis of tumor cells [27,60]. Using molecular docking studies, it was demonstrated that WTHN was able to intercalate into the interfacial cavity between TPX2 and Aurora A, forming hydrogen bonds with surrounding amino acids, thereby developing steric as well as thermodynamic barriers between TPX2 and Aurora A. In the presence of WTHN, the TPX2-Aurora A complex was not able to form properly, or was not able to form at all, so the complex remained catalytically inactive and the mitotic spindle apparatus could not form properly. A similar molecular docking study looked at the interactions of WTHN with survivin, the smallest “inhibitor of apoptosis” protein that is overexpressed in many types of cancers, including glioblastoma and other high-grade gliomas [24,25,26,60,61]. Survivin is able to inhibit apoptosis via the inhibition of caspase activity, as well as by facilitating mitosis through interactions with its BIR5 domain. The docking studies revealed the strong binding affinity of WTHN to the BIR5 domain of survivin, thereby interfering with the ability of survivin to inhibit caspase activity and facilitate mitosis in tumor cells. Mortalin is known to bind to p53 and form a complex, sequestering p53 in the cytoplasm so that it cannot translocate to the nucleus and induce transcriptional activation. In silico and molecular dynamic simulations have demonstrated that WTHN can bind mortalin in the p53 binding site, forming a thermodynamically stable complex [60,62,63]. Withanone binding abrogates the formation of mortalin-p53 complexes, resulting in the increased translocation of p53 into the nucleus and functional reactivation of p53 activity in cancer cells. In addition, molecular docking and in vitro studies suggested that WTHN could also interact at the critical binding site of p21, forming stable hydrogen bonds with Lys and Arg residues at the site [62]. p21 is a p53-dependent inhibitor of cyclin-dependent protein kinases (i.e., CDK-2, CDK-4) and controls the initiation of the S phase in the cell cycle. Cancer cell lines treated with WTHN had evidence for growth arrest that was correlated with an increased expression and activity of p21, as shown by nuclear immunostaining and Western blotting. When cancer cell lines were treated with an ashwagandha leaf extract (i-Extract) or WTHN, there was evidence for the selective killing of cells via the induction of reactive oxygen species (ROS), along with mitochondrial swelling, the loss of mitochondrial membrane potential, and membrane damage [54]. Cell lines were also noted to have cell cycle arrest at G2 and an increased expression of p21.

Withaferin-A, WTHN, and a mixture of the two compounds in a 20:1 ratio (WTHN 10.6 μml/L; WFA 0.53 μml/L; WiNA 20-1) were used to treat various cell lines (osteosarcoma, neuroblastoma, glioblastoma) and nude mouse models [64]. WFA, WTHN, and WiNA 20-1 were all toxic and lethal to the tumor cell lines; however, WTHN and WiNA 20-1 were not toxic to normal cells. If the ratio of WiNA was reduced to 5:1 or 3:1, then it became toxic to normal cells as well. The ability to reduce colony numbers and cell migration was highest when using the WiNA 20-1 compound versus WFA or WTHN alone. In the nude mouse models, there was a strong suppression of the tendency for lung metastases. Three proteins were noted to be downregulated in response to treatment with WiNA 20-1 and WTHN: heterogeneous nuclear ribonucleoprotein K (hnRNP-K), mortalin, and ezrin. hnRNP-K is a multifunctional protein that regulates ERK, MMPs, and VEGF and contributes to cell migration, invasion, and angiogenesis. By downregulating hnRNP-K, WiNA 20-1 and WTHN were able to limit cell growth, migration, and angiogenesis, as well as reduce the tendency for systemic metastases.

Several other studies from the same research group have evaluated the activity of WTHN against EGFR, BCR-ABL, and DNA methyltransferases. In the first study, WTHN was tested against several mutant forms of EGFR (exon 20 insertion, L858R, and exon19del) in molecular docking and molecular dynamic simulations, to see if it could serve as an ATP competitive inhibitor at the active site versus positive controls (e.g., Erlotinib) [65]. Withanone was able to displace and bind at the ATP orthosteric site of all the mutant forms of EGFR. In addition, the binding free energy of WTHN at the ATP site was comparable to Erlotinib and other positive controls. In a similar report, molecular docking studies and molecular dynamic simulations were performed to test if WTHN could affect BCR-ABL signaling, which is constitutively active in chronic myeloid leukemia [66]. Withanone was noted to interact at the catalytic and allosteric sites of the ABL protein, inducing conformational changes and an inhibition similar to the targeted drug Imatinib. In the final study, the authors evaluated WTHN in molecular docking studies and molecular dynamic simulations for the potential to inhibit the DNA methyltranferases (DNMTs)—DNMT1 and DNMT3A [67]. DNMTs are involved in the process of hypermethylating the promoters of tumor suppressor genes (e.g., p16^INK4A^), thereby contributing to neoplastic transformation. Withanone was found to be able to bind to the active sites of DNMT1 and DNMT3A, with the inhibition of activity, as shown by the increased expression of the p16^INK4A^ tumor suppressor gene. The binding affinity of WTHN to DNMT1 and DNMT3A was moderate in comparison to the binding affinity of WFA, which was more robust.

Withanone has demonstrated activity against numerous cancer cell lines in various studies, with different mechanisms of action [24,25,26,60]. It was shown to inhibit cell proliferation in MCF 7 breast cancer cells and U2OS osteosarcoma cell lines through the disruption of the TPX2-Aurora complex, with similar inhibitory effects against A549 lung cancer cells via synergistic activity with cucurbitacin B [27,60]. Withanone was able to arrest growth and induce apoptosis in TIG-1 lung cancer cells, U2OS osteosarcoma cells, and HT1080 fibrosarcoma cells through the increasing activity of p53 [60]. Similar inhibitory affects and induction of apoptosis were noted in osteosarcoma, breast, and fibrosarcoma cell lines through the disruption of the mortalin-p53 complex, with a reactivation of p53 activity. In HUVEC cells, WTHN was able to reduce angiogenesis by inhibiting the tube-forming activity of VEGF [60]. Withanone was able to inhibit the growth and proliferation of breast, colorectal, cervical, osteosarcoma, esophageal, and myelogenous leukemia cells by reducing the oncogenic signaling activity of the ABL1 protein [60]. Breast and colon cancer cells have been shown to respond to treatment with WTHN through the reduced activity of the TPX2, ING1, TFAP2A, and LHX3 genes, along with mitochondrial membrane damage induced by increased ROS stress [60]. In addition, WTHN was able to inhibit osteosarcoma cells through increasing the activity of p21 and inducing cell cycle arrest (see Figure 4).

### 2.5. Withanone: Application to Gliomas

In contrast to WFA, very little research has been performed specifically applying WTHN to glioblastoma or other gliomas in vitro or in vivo [24,25,26,60]. Early studies by Shah and co-workers applied an alcoholic extract (i-Extract) to rat and human glioma cell lines [45]. In addition to i-Extract, the cell lines were also treated with individual components of the extract, including WTHN. All of the treatment arms were able to induce a dose-dependent growth arrest of both cell lines, with a peak effect between 48 and 72 h. The IC_50_ values were lower for the human glioma cells in comparison to the rat glioma cells; the IC_50_ of WTHN was 40 μM. Higher doses of i-Extract and WTHN were able to induce apoptosis in the glioma cell lines. However, when low to moderate doses were applied, cell cycle arrest in the S and G_2_/M phases occurred, along with reduced motility, growth arrest, and a change in phenotype to a more senescent state. In combination studies, WFA (0.01 μM) plus WTHN (5 μg/mL) was the most active in inducing a senescent and more differentiated state. The authors considered ashwagandha and its bioactive phytochemical components as potential treatment options for differentiation therapy in glioblastoma.

Withaferin-A, WTHN, and a mixture of the two compounds in a 20:1 ratio (WTHN 10.6 μml/L; WFA 0.53 μml/L; WiNA 20-1) were used to treat various cell lines, including glioblastoma [64]. WFA, WTHN, and WiNA 20-1 were all toxic and lethal to the glioblastoma cells in a culture. The ability to reduce colony numbers and cell migration was highest when using the WiNA 20-1 compound versus WFA or WTHN alone. Three proteins were noted to be downregulated in response to treatment with WiNA 20-1 and WTHN: heterogeneous nuclear ribonucleoprotein K (hnRNP-K), mortalin, and ezrin. hnRNP-K is a multifunctional protein that regulates ERK, MMPs, and VEGF and contributes to cell migration, invasion, and angiogenesis. By downregulating hnRNP-K, WiNA 20-1 and WTHN were able to limit cell growth, migration, and angiogenesis.

### 2.6. Other Withanolides/Bioactive Compounds: Application to Systemic Cancer and Gliomas

Withanolides other than WFA and WTHN have also been tested against cancer cells in vitro, but to a much lesser extent [24,25,26]. Withanolides-D, -E, and -F, as well as 4β-hydroxywithanolide-E and withalongolide-A, have demonstrated activity against several cell lines, including renal, pancreatic, and colorectal cancers, as well as neuroblastoma. In addition, physalins A, B, F, and H, along with withaphysalins B, F, and N, have displayed strong cytotoxicity against multiple tumor cell lines, including melanoma, fibrosarcoma, lung cancer, colon cancer, prostate cancer, breast cancer, hepatocellular cancer, and leukemia [24,25,26].

In terms of in vivo studies, in a mouse breast cancer xenograft model, 4β-hydroxywithanolide-E (15 mg/kg) was noted to induce a 64.3% reduction in tumor growth [24,25,26]. In a similar breast cancer xenograft model, withanolide-D (5 mg/kg) was demonstrated to reduce tumor growth and angiogenesis. Withanolide-D has also been tested against nude mice with leukemia xenografts (10 mg/kg), with an 80% reduction in tumor burden. In nude mouse models of hepatocellular carcinoma and colorectal cancer, 4β-hydroxywithanolide-E was able to induce a marked reduction in tumor volume in both tumor types. Physalin A was tested against xenograft models of non-small cell lung cancer (40 or 80 mg/kg) and was noted to significantly reduce tumor growth. In an MDA-MB-231 breast cancer nude mouse xenograft model, tubocapsanolide-A was able to reduce tumor growth and inhibit the tendency for lymphatic invasion.

None of the above-mentioned less commonly studied withanolides has been specifically applied to glioblastoma or other gliomas, except for WTHA (see Figure 2D), which was evaluated by Shah and colleagues [45]. Withanolide-A was tested against C6 (rat glioma) and YKG1 (human glioma) cell lines in vitro. Similarly to what was reported above, treatment with WTHA was able to induce the significant growth arrest of both cell lines in a dose-dependent manner. The IC_50_ values for WTHA ranged from 20 to 35 μg/mL. When high doses of WTHA were used, apoptosis was activated in the glioma cell lines. However, when low to moderate doses were used, there was cell cycle arrest in the S and G_2_/M phases (see Figure 4), as well as reduced motility, growth arrest, and a change in phenotype to a more senescent state.

## 3. Conclusions and Future Considerations

In recent years, Ayurvedic physicians and researchers have begun an attempt to correlate the ancient philosophy and tenets of Ayurveda with modern medicine, molecular biology, and the biosciences [68,69]. For example, Gupta and colleagues have performed a “scoping review” of modern assessment tools for determining the *Prakriti* and Dosha balance of an individual [70]. Some older methods, such as pulse diagnosis and plethysmography, have been shown to be too subjective, with poor inter-rater reliability. Various types of detailed questionnaires seem to be the most accurate and reliable method of determining *Prakriti* at the moment, but more research is needed. Other researchers are investigating the new field of “Ayurgenomics”, which attempts to correlate the Doshas and *Prakriti* with modern medical genetics and molecular biology [71,72]. Preliminary studies are starting to correlate *Prakriti* types (*Vata*, *Pitta*, *Kapha*) with specific genetic parameters, such as HLA DRB1 types, genomic-wide expression levels, DNA methylation signatures, and various disease conditions, with many studies able to discern differences between the Dosha types [71]. In addition, Sharma and Wallace propose that the *Prakriti* of an individual can be correlated with their “Genotype (DNA)”, with *Vata* correlating with mRNA, *Pitta* correlating with tRNA, and *Kapha* correlating with proteins [72]. Epigenetic changes are then correlated with *Karma* (Action), resulting in the phenotype of an individual, which is affected by lifestyle and behavior, diet and digestion, stress, and environmental factors. Finally, another group has been attempting to correlate *Vata*, *Pitta*, and *Kapha* individuals with patterns of functioning of the six major areas of the nervous system: the prefrontal cortex, reticular activating system, autonomic nervous system, enteric nervous system, limbic system, and hypothalamus [73]. A model was developed that was able to differentiate neural function in these six brain areas based on Dosha type. The authors believe this model can be used moving forward to assist in further neurological research in this area using neuroimaging, EEG, autonomic measures, psychological measures, and other techniques.

It is apparent from the overview of Ayurveda and ashwagandha in Sections I and II that many of the plants, herbs, and herbal formulations that have been used for millennia by Ayurvedic physicians have significant activity against numerous human illnesses, including inflammation, digestion, diabetes, depression, and many others, including cancer. More recent ethnopharmacological and phytochemical analyses have been able to clarify the likely bioactive compounds that are mediating these beneficial effects—WFA, WTHN, other withanolides, etc. A further analysis of ashwagandha and its various herbal formulations will be important to determine all of the active compounds, and if they could be more specifically applied to cancer treatment. In addition, it may be helpful to also consider a “Network Pharmacology” approach during these investigations, since many of the bioactive compounds and/or formulations seem to be modulating multiple targets simultaneously.

Withaferin-A has been evaluated in detail in terms of its ability to inhibit tumor growth in cultured cancer cells, as well as in in vivo xenograft models, including glioblastoma. The mechanisms of action of WFA against tumor cells have also been clarified in detail, affecting numerous pathways that mediate cell proliferation, the cell cycle, internal signal transduction pathways, and apoptosis simultaneously. However, for WTHN, WTHA, 4β-hydroxywithanolide-E, and the many other bioactive compounds that are present in the ashwagandha plant, the pre-clinical evaluation of anti-cancer activity is still very preliminary and immature. For all of these compounds, there is a need for additional in vitro testing against a broad panel of cancer types, including glioblastoma and other gliomas. For those compounds that have a positive signal for activity in cell cultures, in vivo testing in mouse xenograft models will also be necessary and important.

Clinical trials have recently started to apply Ayurvedic herbs and preparations to human subjects for the treatment of various diseases, as well as for pharmacokinetic investigations. For example, ashwagandha and withanolides have recently been applied to numerous systemic diseases and conditions, including schizophrenia, STAR (strength training adaptations and recovery), idiopathic male infertility, subclinical hypothyroidism, mild cognitive impairment, and body weight management under chronic stress [14,15,16,17,18,19]. Much of the early evidence suggests that these herbs and preparations demonstrate a positive effect on these illnesses and have been well-tolerated overall. Furthermore, pharmacokinetic studies have been performed in healthy volunteers using an ashwagandha root extract after ingesting a 500 mg capsule [74]. The results included a C_max_ for WTHA and WFA of 2.926 ± 1.317 and 2.833 ± 0.981, respectively, along with a T_max_ of 1.361 ± 0.850 and 0.903 ± 0.273, respectively. Additional studies will be needed to delineate the efficacy and tolerability of ashwagandha, WFA, WTHN, and their various preparations in other conditions and medical diseases.

The current worldwide standard of care for the treatment of GBM and other malignant gliomas is to perform a maximal surgical resection whenever possible, followed by 6000 cGy of radiotherapy (RT) in 30 fractions, along with concomitant daily oral Temozolomide (TMZ) chemotherapy (75 mg/m^2^/day × 42 days) [75,76]. After the completion of chemo-RT, the patient is then treated with adjuvant chemotherapy with TMZ (150–200 mg/m^2^/day) for 5 days every 28 days, for at least six cycles. Using this approach with the addition of TTFs, the median survival in recent phase III trials is in the range of 18 to 24 months. Other than this approach, there are only a few FDA approved treatment options, including the use of TTFs and Bevacizumab—a monoclonal antibody targeted to VEGF [57,75,77].

There are many ongoing clinical trials for patients with GBM and high-grade gliomas, designed to find a drug or compound that might add progression-free and overall survival time to what has been attained with TMZ-based chemo-RT and adjuvant TMZ ± TTFs. Based on the review above of ashwagandha and the phytochemical analyses of several of its most bioactive components, there is significant evidence that several of these compounds and formulations have anti-cancer potential (e.g., AshwaMax, ASH-WEX, WFA, WTHN). In addition, they are natural products that have been used for millennia and have proved to be well tolerated in the traditional setting, as well as in early clinical trials. In the near future, it will be critical to apply these more specifically to the treatment of GBM and other gliomas. Additional in vitro studies will be needed, especially in more sophisticated model systems, such as GBM organoid cultures, which more closely resemble the three-dimensional structure and immune environment of the human tumor [78]. Thus far, there have been very few phase I or II clinical trials with an Ayurvedic-related compound or herbal extract in patients with GBM, medulloblastoma, or any other type of malignant brain tumor. For example, there is a bioavailability trial of curcumin in GBM patients who require surgical resection that has been launched in Germany (NCT01712542). There are no current clinical trials listed for ashwagandha, WFA, or WTHN for the treatment of GBM or other brain tumors on ClinicalTrials.gov. It will be important to bring ashwagandha and some of the other Ayurvedic compounds into clinical trials for GBM patients, since the pre-clinical and animal studies for several of them have been so compelling—in particular WFA—including reports suggesting synergistic interactions with TMZ and TTFs. The addition of one of these products to TMZ during chemo-RT and adjuvant monthly chemotherapy in newly diagnosed GBM would be a reasonable first step in a small pilot study or a larger phase I trial. Another option would be to consider adding WFA or an ashwagandha product to TTFs in the setting of recurrent or progressive GBM. In addition to the hope for an anti-cancer effect that might be synergistic with RT/TMZ and monthly TMZ (or TTFs), there is also the possibility that the addition of these formulations or compounds might have other clinical benefits as well, such as less fatigue, reduced GI upset, better digestion, improved immune strength, and less RT-induced inflammation. During the adjuvant phase of TMZ treatment, which can last 6–12 months in many patients, the use of ashwagandha formulations and compounds could add significantly to patient tolerability of therapy, with an overall improved quality of life.

Major drawbacks and limitations when considering these potential studies will include the consistency and dependability of the manufacturing process for ashwagandha and its related products, with well-defined concentrations of WFA, WTHN, WTHA, and other withanolides. Another important issue will be to make sure that the concentrations of heavy metals and other contaminants are within international standards. Other considerations will include the potential for interactions of ashwagandha and its phytochemicals with ongoing medical and oncological therapies, particularly the modification of liver-mediated drug metabolism. Finally, issues related to poor bioavailability and attaining adequate concentrations of phytochemicals in the nervous system will also have to be addressed.

## Figures and Tables

**Table 1:** Partial listing of traditional uses of ashwagandha.

Plant Parts	Ethnomedicinal Use	Preparation	Route
Root	Anemia	Root powder	Oral
Whole plant	Arthritis, anxiety, insomnia	Whole plant	Oral
Leaves	Weight loss	Raw leaves	Oral
Leaves and roots	Diabetes	Leaf infusion and root powder	Oral
Leaves	Memory enhancer	Fresh leaves	Oral
Root	Joint pain	Stem juice	Oral
Root	Anti-tumor	Root powder	Oral
Root	Aphrodisiac, impotency	Root powder	Oral
Whole plant	Menstruation	Powder	Oral
Root tuber	Skin cancers	Paste	Topical
Bark	Piles	Decoction	Topical
Tubers	Tumors	Paste	Topical
Rhizome	Nervous disorders	Decoction	Oral
Tuber	Healing wounds	Paste	Topical
Root	Fatigue, poor appetite	Decoction	Oral
Leaves	Wounds and burns	Paste	Topical
Root	Seizure activity	Root juice	Oral
Roots and leaves	Female sterility	Root/leaf decoction	Oral
Whole plant and flowers and fruits	Breast cancer	Powder	Oral
Leaves and fruits	Digestive problems	Powder	Oral
Whole plant and roots	Male sterility	Plant/root powder	Oral

Data derived from references [10,11,12,13].

**Table 2:** Listing of the different classes of phytochemicals present in ashwagandha.

Steroidal Lactones	Alkaloids	Flavonoids	Salts	Steroids	Nitrogen-Containing Compounds
Withaferin-A	Withanine	Kaempferol	Cuscohygrine	Cholesterol	Withanol
Withanone	Withananine	Quercetin	Tropine	Sitoinosides	Aomnitol
Withanolide-A	Withasomnine		Pseudotropin	Diosgenin	Somnisol
E, F, G, H, I	Somniferine		Anahygrine	Sigmasterol	
J, K, L, M	Somniferinine		Anaferine	β-sitosterol	
	Nicotine			Stigmastadien	
	Tropeltigloate				

Data derived from references [10,11,12,13].

**Table 3:** Summary of application of ashwagandha and bioactive compounds to gliomas.

ASHWAGANDHA/WITHAFERIN-A (WFA)		
Herb Preparation	Tumor Type	Anti-Cancer Effect
Alcoholic extract leaves, WFA, withanolide-A, withanone [43]	C6 rat glioma and YKG1 human glioma cell lines	Induce growth arrest; induce apoptosis; cell cycle arrest; WFA lowest IC_50_
Aqueous extract leaves [44]	C6 rat glioma and YKG1, U118MG, A172 human glioma cell lines	Reduce proliferation; induce apoptosis; inhibit cell cycle; induce differentiation
ASH-WEX aqueous extract [45]	C6 rat glioma intracranial xenografts	Reduced pNF-κB, pAkt, VEGF, cyclin D1, and Bcl-xl; enhanced apoptosis; cell cycle arrest G_1_/G_0_; tumor shrinkage
WFA [48]	GL26 murine and U87, U251 human glioma cell lines	Dose-dependent shift cell cycle; G_2_/M arrest: WFA—0.5–1.5 µM; induce apoptosis; reduce Akt and mTOR; reduce EGFR and cMET
WFA [49]	TMZ-resistant U251, U87 human glioma cell lines	Reduce proliferation; cell cycle arrest G_2_/M; induce apoptosis; reduce EGFR and cMET; reduce MGMT
WFA [50]	C6 rat glioma cell lines	Induce apoptosis; induce caspase-3, -9; increase Bax; reduce Bcl2; reduce TNF-α and NF-κB
WFA and AshwaMAX [51]	U87, GBM2, GBM39 human glioma cell lines; mouse xenograft model	Reduce proliferation, reduce nanospheres—WFA 0.25–0.31 µM, AshwaMAX 2.1–14.8 µg/mL; xenografts inhibited x 3–4 weeks
WFA and AshwaMAX, tumor treating fields (TTFs) [52]	U87, GBM2, GBM39 human glioma cell lines	Synergistic effect of WFA and TTFs; reduce growth cell lines and neurospheres
WFA [53]	U251, U87 human glioma cell lines and nude mouse xenografts	Inhibit cell growth; increase apoptosis; increase Bim, Bad; cell cycle arrest G_2_/M; shrink xenografts
**WITHANONE**		
**Aqueous extract leaves** [44]	**C6 rat glioma and YKG1, U118MG, A172 human glioma cell lines**	**Reduce proliferation; induce apoptosis; inhibit cell cycle; induce differentiation**
Withanone [54]	C6 rat glioma and YKG1, A172 human glioma cell lines	Reduce hnRNP-6, mortalin, and ezrin; reduce VEGF
**WITHANOLIDE-A**		
**Aqueous extract leaves** [44]	**C6 rat glioma and YKG1, U118MG, A172 human glioma cell lines**	**Reduce proliferation; induce apoptosis; inhibit cell cycle; induce differentiation**

## Data Availability

No new data were created or analyzed in this study. Data sharing is not applicable.
